# Neonatal Screening for Congenital Adrenal Hyperplasia in Indian Newborns with Reflex Genetic Analysis of 21-Hydroxylase Deficiency

**DOI:** 10.3390/ijns9010009

**Published:** 2023-02-21

**Authors:** Jayakrishna Tippabathani, Venu Seenappa, Alagupandian Murugan, Nagaraja Mahishi Phani, Mahesh H. Hampe, Giridharan Appaswamy, Prakash Sadashiv Gambhir

**Affiliations:** 1 Lifecell International Pvt Ltd., Vandalur, Kelambakkam Road, Chennai 600127, India; 2 Chief Medical Scientist, Lifecell, West Regional Lab, Pune 411048, India

**Keywords:** congenitaladrenal hyperplasia (CAH), neonatal screening, *CYP21A2* reflex genetic testing, 21-OH hydroxylase deficiency

## Abstract

Congenital adrenal hyperplasia (CAH), screened for in neonates, is the second most common endocrinopathy after congenital hypothyroidism.Newborn screening for CAH due to CYP21A2 deficiency is performed by immunologic assay for 17-hydroxyprogesterone (17-OHP). The second-tier test for confirmation of diagnosis is carried out on recall venous blood sample from screen positives measuring 17-OHP, or other metabolites of steroid metabolism by liquid chromatography–tandem mass spectroscopy. However, as steroid metabolism is dynamic, it can affect these parameters even in the recall sample of a stressed neonate. Moreover, there is some time delay in recalling the neonate for repeat testing. Reflex genetic analysis of blood spot from the initial Guthrie cards of screen positive neonates, if used for confirmatory testing, can avoid this time delay as well as the effect of stress on steroid metabolism. In this study, we used Sanger sequencing and MLPA in a reflex manner for molecular genetic analysis to confirm *CYP21A2*-mediated CAH. Out of 220,000 newborns screened, 97 were positive on the initial biochemical screen, of which 54 were confirmed true positives with genetic reflex testing, giving incidence of CAH as 1:4074. Point mutations were more common than deletions, indicating that Sanger sequencing should be used ahead of MLPA for molecular diagnosis in India. Amongst the variants detected, the most common was I2G-Splice variant (44.5%), followed by c.955C>T (p.Gln319Ter) (21.2%); Del 8 bp and c.-113G>A were detected with frequencies of 20.3% and 20%, respectively. In conclusion, reflex genetic testing is an effective strategy for identifying true positives in CAH screening in neonates. This will obviate need for recall samples and also aid effective counselling and timely prenatal diagnosis in the future. In Indian newborns, as point mutations are more common than large deletions, Sanger sequencing should be the initial method of choice for genotyping, ahead of MLPA.

## 1. Introduction

Congenital adrenal hyperplasia (CAH) is an autosomal recessive genetic disorder caused by mutations in seven genes encoding the enzymes involved in adrenal steroidogenesis. Theenzyme encoding genesinclude 21-hydroxylase (21OH), 11β-hydroxylase (11βOH), 17α-hydroxylase (17OH; also known as 17,20-lyase), 3β-hydroxysteroid dehydrogenase type 2 (3βHSD2), steroidogenic acute regulatory protein (StAR), P450 cholesterol side-chain cleavage enzyme (SCC), and P450 oxidoreductase (POR). In 95% of cases, CAH results from mutations in the *CYP21A2* gene encoding for the enzyme 21-OH hydroxylase [1,2]. 21-OH hydroxylase deficiency causes impairment in the production of cortisol and aldosterone and androgen excess. Hormonal imbalances manifest phenotypically under two broad categories: classical CAH and non-classical CAH. The classical CAH is alife-threatening salt-wasting (SW) type which may present as an acute emergency in neonates and simple virilizing (SV), causing ambiguity in genitalia in females [3,4,5]. 21-OH deficiency can be effectively treated by hormone replacement therapy. The incidence rate of CAH worldwide is about 1 in 15,000 and 20,000 live births.InIndia, CAH is more common, with the largest study reportinganincidencerateof 1 in 5762 [6,7]. With due consideration to the incidence and efficacy of treatment, thewmajority of newborn screening programs have CAH as an essential component along with congenital hypothyroidism and other IEMs [8].

17-OHP (17 Hydroxyprogesterone) is used as the marker for CAH in newborn screening. Efficient screening programs aim to have excellent sensitivity and minimal false positivity. In the case of CAH, this is complicated because of the dynamic nature of steroid metabolism, i.e., it being influenced by birth weight, gestation, antenatal administration of glucocorticoids, day of life, and neonatal stress. To avoid anxiety throughfalse positives and to confirm true positives, second-tier testing on primary or second sample post recall is employed with other steroids measured with LC-MS/MS (Liquid chromatography-tandem mass spectrometry)instead of 17-OHP level alone. This second-tier testing with LC-MS/MS has indeed been shown to reduce the false positive rate and improve the positive predictive value. Therefore, Clinical practice guidelines of Endocrine society recommend second-tier testing with LC-MS/MS over other methods [9,10,11,12]. However, these analytes, again being part of steroid metabolism, can also be potentially affected by stress.

Currently, good quality DNA suitable for genetic testing can be easily retrieved from dried blood spots on Guthrie cards. This DNA can be utilized to achieve molecular diagnosis of CAH.The advantage of reflex genetic testing for mutations from the same card is that stress or other modifiers cannot influence it and a repeat sample is not required.

The current study was planned to evaluate reflex genetic testing for *CYP21A2* mutations from the DBS from Guthrie cards as a confirmatory test in screen positives in a large multianalyte screening program in Indian newborns.

## 2. Materials and Methods

### 2.1. Study Participants

The study participants consisted of 220,000 newborns from different birthing centers throughoutIndia undergoing voluntary multianalyte neonatal screening. Informed consent was obtained from parents. Samples were collected in the majority of cases after 48 h of life by heel prick on a Guthrie card on which demographic details of the newborn were captured. 17-OHP hormone levels were measured using GSP^®^ instruments, specifically the GSP Neonatal 17-OHP progesterone kit with Auto DELFIA^®^ (Perkin Elmer, Turku, Finland). DNA was isolated from screen positive Guthrie cards by using MagMAXDNAmulti-sample kit.

In the initial phase of study, screen positive samples with values more than 140 nmol/L were first subjected to MLPA and those with values less than 140 nmol/L were subjected to Sanger sequencing. This protocol was based on the consideration that large deletions will produce more functional impairment of *CYP21A2 gene* resulting in higher 17-OHP levels. However, in the course of study, after first few true positive results, it became evident that point mutations were much more common than deletions. Thereafter, Sanger sequencing becamethe initial method used for all screen positives. If the initial technique did not yield an informative result, the second method was opted for.

### 2.2. Sanger Sequencing

*CYP21A2* gene-specific amplification was performed and followed by sequencing of the *CYP21A2* gene with specific primers using ABI 3500 Genetic Analyzer as previously described [13,14]. The result was analyzed using the Mutation Surveyor software version 5.1 (Soft genetics, State College, PA, USA).

The variants were predicted and classified according to ACMG (American College of Medical Genetics and Genomics) guidelines. Observed variants were named according to the Human Genome Variation Society nomenclature guideline (http://www.hgvs.org/ (accessed on 10 November 2022)) [15].

### 2.3. Multiplex Ligation-DependentProbe Amplification (MLPA)

Large deletions in the CYP21A2 were screened by MLPA using the P050-CAH kit (MRC, Holland). The results were analyzed using Coffalyser. NET software (MRC-Holland, Amsterdam, The Netherlands).

## 3. Results

A total of 97 newborns out of 220,000 were identified as screen positives with screen positivity rate of 0.044%.Out of these 97 screen positives, 39 had homozygous status for *CYP21A2* mutations, while 15 had compound heterozygous status.

### Molecular Confirmation

Supplementary Table S1 gives details of pathogenic variants detected in each case by the MLPA and the Sanger sequencing methods. Among the 97 neonates genotyped for pathogenic variants in the *CYP21A2* gene, causative variants for CAH in *CYP21A2* were detected in 54 infantsby both Sanger sequencing and MLPA methods. A total of 8 different types of point mutations and 4 large deletions were detected inin 54 neonates Point mutations occurred at 92.6%,whilelarge deletion variants occurred at a frequency of 7.44%.The most prevalent pathogenic variant identified in the current study was I2G-Splice (c.293-13A/C>G) found at a distribution frequency of 44.5%. Del 8 bp (c.332_339 delGAGACTAC), and promotor mutation c.-113G>A showed 20.3% (12 neonates) and 20% (11 neonates) of frequency distribution, respectively. The mutation p.Gln319Ter (c.955C>T) was found at an occurrence rate of 21.2% (23 neonates). A combination of p.Gln319Ter with other variants was detected in nine neonates and a combination of 8 bp Del with other variants was also identified in nine neonates (Table 1 and Supplementary Table S1).

Concerning genotype status, in 39 (72.22%) neonates, the causative variants occurred in a homozygous condition, and in 15 (27.77%) neonates in a compound heterozygous condition. The frequent pathogenic variant detected in the homozygous state was I2G splice (14 infants) and Del8bp (10 infants). The most commonly identified point mutation was p. Gln319Ter in seven neonates in the homozygous state, and in nine neonates in the compound heterozygous state. Four different types of deletion associated variants were found: deletion of Promotor to Exon 7 (homozygous state) was identified in a single case, deletion of promotor to Exon 3 with c.1069C>T (p.Arg357Trp) (Compound heterozygous state) was identified in another case; similarly, deletion of the promoter to exon 6 with p.Arg357Trp (compound heterozygous state) was identified in the other case. A large deletion associated with E6 cluster variants (compound heterozygous state) was identified in one of the cases. Novel mutant c.1118+2T>C was found in intron 8 in one of the newborns; to our knowledge, this splice site variant has not been reported previously. In silico analysis predicted the novel mutant to be highly pathogenic as it lies in the +2 intronicposition.Toassess the impact of variants three in silico tools—spliceAI, maxEntScan, and CADD—were used to predict the penetrant effect of the novel variant c.1118+2T>C identified in the *CYP21A2* gene, and it is predicted to be thedeleterious and fully penetrant as the variant lies in the essential dinucleotide of the splice junction.

## 4. Discussion

The objective of Neonatal screening is to identify treatable disorders inthepre-symptomatic stage so that they can be managed most effectively for a better outcome. CAH is the second most common endocrinopathy screened in neonates after congenital hypothyroidism. Neonatal screening for CAH in the first week is probably even more crucial than CH, as undetected and untreated babies affected with the salt loosing variety may have a crisis within a few days of birth. However, in comparison to congenital hypothyroidism, CAH screening for *CYP21A2* is more complex as the steroid metabolism has many variables affecting it and false positive results increasing parental anxiety are likely unless a prompt and effective second-tier testing to confirm true positives isin place [16].

The present study included 97 newborns identified with elevated 17-OHP levels in a cohort of 220,000 newbornsthroughout India volunteeredmultianalyte neonatal screening. MLPA was the initial test chosen for newborns with 17-OHP >140 nmol/L, while Sanger sequencing was the first technique chosen for screen-positive samples below 140 nmol/L. This strategy was adopted to shorten the reporting time of the confirmatory molecular test and also to reduce the costs. However, when initial data showed that point mutations were more common than deletions, Sanger sequencing was used as the initial method for molecular characterization of all screen positives.

Screen positivity for 17-OHP was 97/220,000, i.e., 0.044%, while out of these, 54 cases were true positives on reflex genetic screening, giving a prevalence of1:4074 births for 21 hydroxylase deficiency. This is the largest newborn screening study reported from India. An ICMR pilot project which had nearly one lakh newborn has reported overall prevalence of CAH as 1:5762 [17,18]. Our study and the ICMR study have demonstrated that CAH is much more common in India than rest of the world and should be included in the basic panel for NBS.

Important considerations for a newborn screening program are the detection rate, screen positive rate, and the positive predictive value of the test. In past newborn screening studies with sample sizes of more than 10,000 newborns from India, the screen positivity has ranged from 0.03% to 0.13% [16]. The positive predictive value in these studies has ranged from 16.8% to 100% [16]. In the present study, the screen positivity was 0.044%, while the positive predictive value was 55.7%.

Confirmatory testing for the screen positive newborn is usually carried out on a venous recall sample either by immunoassays for hormones or by steroid profiling using the ratios of steroid metabolites [19]. Recalling and sampling may take time and some screen positives may not be available for retesting. In the present study, DNA extracted from the initial Guthrie card was utilized for genetic testing; thus, recalling the baby for second sample was not required. Previous studies fromIndiahave reported that tracing patients was difficult and only 80% of screen positives could be tested even in a well-planned study [17]. Reflex genetic testing will therefore be ideal in India as recall is not required. Moreover, genotype is not affected by stress, unlike the other metabolites of steroid metabolism.

As the salt wasting variety of CAH can manifest with serious symptoms in the first few days of life, the turnaround time for the confirmatory test is crucial. In our study, we had informed the concerned clinician of the positive screening result, who then initiated the biochemical testing on recall sample at his end. Mean turnaround time for reflex molecular confirmation was 8.2 days, while for 50% of confirmed cases it was less than a week. This is comparable to the reporting time in some newborn screening programs in the developed world [9]. In seven cases, turnaround time was more than 10 days, the longest being 15 days. These cases were in the initial phase of study where MLPA was used as the first test. Using Sanger sequencing in the first place would have avoided this delayed confirmation. One of these babies had salt loosing onset on day 8 of life. In some newborns, molecular confirmation may be delayed, especially when first technique does not yield a positive result. Therefore, we feel that reflex testing of DBS on primary Guthrie card should be used with confirmatory biochemical testing on the recall sample. This will give a comprehensive genotype and phenotype correlation. Moreover, those who default on recall can be actively traced. The quickest result will lead to prompt initiation of therapy in affected babies.

As far as the genotyping of the affected newborns was concerned, 72.22% were homozygous (39/54), and 27.77% (15/54) were compound heterozygotes.

The frequency of large deletions detectable by MLPA alone was (7.4%). Sanger sequencing was thus able to achieve molecular diagnosis in 92.59% (Supplementary Table S1) of cases that had point mutations as well as small deletions. Thus, Sanger sequencing is the method of first choice to reach molecular diagnosis in India.

The I2G-splice mutation detected at higher percentages (44.5%) in the present study is somewhat comparable with the frequency in Denmark (33.8%), and the frequency observed is similar to other studies conducted in the Indian populations [20,21,22]. Compared to most other populations, the pathogenic variant p.Gln319Teroccurred at a higher frequency in the current study. I2G splice mutation of *CYP21A2* is commonly distributed among most world populations at a higher frequency compared to other disease-causing variants [20]. The mutations that occurred more frequently, next to I2G splice, include p.Gln319Ter (21.2%) and p.Gly111Valfs*21. Similarly, such observations of the occurrence of p.Gln319Ter and p.Gly111Valfs*21 are reported in other studies conducted in the Indian population [20,21,22]. Compared to other world population distribution of p.Gln319Ter, the mutation occurred at a higher frequency (21.2%) in the present Indian neonates [20]. The mutation p.Val282Leu is found in greater frequency in most other aucasian populations but is absent in the current Indian newborn screening study and other studies conducted in the Indian population [23,24]. Variant p.Val282Leu is usually associated with the non-classic form which is not the primary target of newborn screening for CAH

A study of 310 symptomatic CAH cases from India has reportedI2G-spliceas the most common mutation, followed by the 30-kb deletion, p.Ile173Asn, p.Gln319Ter, and p.Arg357Trp [25]. This is similar to study where I2G splice was found at high frequency, followed by p.Gln319Ter and p.Arg357Trp, suggesting point mutations are commonly found in Indian cohorts followed by 30 Kb deletions.

A novel homozygous splice site variant c.1118+2T>C at intron 8 was identified in one of the newborns. Functional characterization of novel variant using bioinformatics tools pointed to an altered amino acid sequence, indicating that the novel variant may be highly pathogenic, affecting the function.

In the current study, the majority of the mutations was carried in the homozygous condition (72.22%), and this percentage is higher compared to the countries such as Serbia 13%, China 16.1%, USA 21%, Tunisia 24%, the Netherlands 28.3%, Croatia 33%, Romania 33.3%, and Brazil 39% [23,24]. This can be attributed to the higher frequency of consanguineous marriages, especially in southern states of India.

For establishing a molecular diagnosis of CAH, the two commonly used techniques are MLPA to detect deletions/duplications, and Sanger sequencing, which can detect point mutations and small deletions as well. The present study shows that point mutations are much more common in Indian newborns. This mandates that Sanger sequencing should be the first technique that should be used for molecular diagnosis. If mutations are not detected by Sanger sequencing, MLPA can be employed. This strategy will reduce the costs as well as improve the reporting time.

Screen positives identified by elevated 17-OHP levels require confirmation by demonstration of same on a repeat sample or by steroid profiling by MSMS on arepeat sample. This requires communication with the clinician and recall of the baby’s transport of the sampleand the test leading to delay in diagnosis. Moreover, though repeat 17-OHP of the venous sample is widely available, steroid profiling by MSMS is not easily available in India [16]. Molecular confirmation from the same initial Guthrie card does not require recall of the patient and resampling. Moreover, unlike the steroid profile genotype, it is constant and is not affected by metabolic alterations or stress. Administration of glucocorticoids to the mother or mutations producing less dysfunctional enzyme may produce a less pronounced rise in 17-OHP or disturbance in steroid profiling and this may be missed on biochemical testing. Second-tier genetic testing can pick true positive cases in these situations also.

Steroid profiling has a distinct advantage over genotyping in identifying other causes of CAH apart from 21 hydroxylase deficiency. It also has a better turnaround time and is less costly than genotyping for *CYP21A2* alone, especially when molecular characterization is not achieved by the initial test. However, genotyping scores over steroid profiling as mutation identification in counseling the couple, especially from the point of future pregnancy planning, will make a timely prenatal diagnosis easily achievable. The strength of our study is the large pan-India newborn sample size in which screen positives have been identified and the reflex molecular screening strategy which uses both Sanger sequencing and MLPA. We have demonstrated that reflex screening for CAH using molecular diagnosis is an effective and possibly ideal strategy, especially in India.

## Figures and Tables

**Table 1:** Frequency distribution of different *CYP21A2* mutations.

Type of Mutation	Number of Cases (Number of Alleles Carrying That Mutation)		Number (%) of Mutant Alleles
	Homozygous	Heterozygous	
I2G-Splice	21(42)	7(7)	49 (44.5%)
c.-113G>A	11(22)		22 (20%)
Del 8 bp	10(20)	2(2)	22 (20.3%)
p.Arg357Trp	2(4)	2(2)	6 (5.5%)
p.Ile173Asn	1(2)		2 (1.8%)
p.Leu308PhefsTer6	1(2)	2(2)	4 (3.7%)
p.Gln319Ter	7(14)	9(9)	23 (21.2%)
p.Arg355Hi		1(1)	1 (0.92%)
p.Gly111Valfs		1(1)	1 (0.92%)
p.Ala14Ser		1(1)	1 (0.92%)
Large Deletions	1(2)	3(3)	5 (4.62%)
Cluster E6		2(2)	2 (1.8%)
c.1118+2T>C,	1(2)		2 (1.8%)
Total Detected Mutations	55(110)	30(30)	
Total Cases	54(108)		

## Data Availability

The data presented in this study are available on request from the corresponding author. The data are not publicly available to protect patient privacy.

## Supplementary Materials

The following supporting information can be downloaded at: https://www.mdpi.com/article/10.3390/ijns9010009/s1, Table S1: Clinical data of affected newborns: Screening 17-OHP Levels and CYP21A2 Mutations.
