# Supplementary material for: Neonatal Screening for Congenital Adrenal Hyperplasia in Indian Newborns with Reflex Genetic Analysis of 21-Hydroxylase Deficiency
Source: Int J Neonatal Screen. 2023 Feb 21;9(1):9. doi: 10.3390/ijns9010009 (PMC10053538; doi:10.3390/ijns9010009)
Supplement: Supplementary file 1 [file IJNS-09-00009-s001.zip › IJNS-2170390-supplementary.pdf]

Case No	17-OHP	Detection Method	Variant details	Zygosity
Case-1	>287.3nmol/L	M+S	c.923dup p.Leu308PhefsTer6 & c. 293-13 A>G	Compound heterozygous
Case-2	266.9nmol/L	S	c.955C>T p.Gln319Ter	Homozygous
Case-3	>266.9nmol/L	M+S	c.955C>T p.Gln319Ter	Homozygous
Case-4	>266.9nmol/L	M+S	c.955C>T p.Gln319Ter	Homozygous
Case-5	>266.9nmol/L	M+S	c.923dup p.Leu308PhefsTer6	Homozygous
Case-6	>266.9 nmol/L	S	c.955C>T p.Gln319Ter	Homozygous
Case-7	222.3 nmol/L	M	c.293-13 A/C>G & c.-113G>A.	Homozygous
Case-8	>266.9 nmol/L	M	c.-113G>A	Homozygous
Case-9	88.3 nmol/L	S	c.293-13 A/C>G	Homozygous
Case-10	>296.9nmol/L	M+S	c.1069C>T p.Arg357Trp	Homozygous
Case-11	>313.2 nmol/L	M+S	c.1069C>T p.Arg357Trp	Homozygous
Case-12	70.5nmol/L	S	c.518T>A p.Ile173Asn	Homozygous
Case-13	>265.5 nmol/L	M	c.293-13 A/C>G, c.332_339del 8bp Del & c.-113G>A	Homozygous
Case-14	>265.5 nmol/L	M	c.293-13 A/C>G & c.-113G>A	Homozygous
Case-15	264.9 nmol/L	M+S	c.955C>T p.Gln319Ter & c.293-13 A/C>G	Compound heterozygous
Case-16	>287.3 nmol/L	S	c.955C>T p.Gln319Ter & c.293-13 A/C>G	Compound heterozygous
Case-17	>287.3 nmol/L	M	c.332_339del 8bp Del & c.-113G>A	Homozygous
Case-18	>287.3 nmol/L	S	c.955C>T p.Gln319Ter	Homozygous
Case-19	>287.3 nmol/L	M+S	c.955C>T p.Gln319Ter	Homozygous
Case-20	>287.3 nmol/L	M	c.293-13 A/C>G, c.332_339del 8bp Del & c.-113G>A	Homozygous
Case-21	>287.3nmol/L	M+S	c.955C>T p.Gln319Ter & c.923dup p.Leu308PhefsTer6	Compound heterozygous
Case-22	>287.3 nmol/L	S	c.955C>T p.Gln319Ter,c.293-13 A/C>G	Compound heterozygous
Case-23	164.4 nmol/L	S	c.293-13 A/C>G	Homozygous
Case-24	>295.5 nmol/L	M+S	Large Deletion (Promoter to Exon 6 of CYP21A2 gene) & E6 cluster variants	Compound heterozygous
Case-25	>295.5 nmol/L	M	c.293-13 A/C>G, c.332_339del 8bp Del & c.-113G>A	Homozygous
Case-26	>295.5 nmol/L	M+S	c.955C>T p.Gln319Ter,c.293-13 A/C>G	Compound heterozygous
Case-27	>295.5 nmol/L	M+S	c.955C>T p.Gln319Ter , c.332_339del 8bp Del & p.Gly111Valfs*	Compound heterozygous
Case-28	>268.3 nmol/L	M	Deletion of 5' UTR to Exon 7 of CYP21A2 gene	Homozygous
Case-29	>303.7 nmol/L	M	c.332_339del & 8bp Del c.-113G>A	Homozygous

Case-30	110.5 nmol/L	S	c.955C>T p.Gln319Ter & c.1069C>T p.Arg357Trp	Compound heterozygous
Case-31	>303.7 nmol/L	M+S	c.955C>T p.Gln319Ter & c.1064G>A p.Arg355His	Compound heterozygous
Case-32	>303.7 nmol/L	M	c.293-13 A/C>G	Homozygous
Case-33	>303.7 nmol/L	M	c.293-13 A/C>G	Homozygous
Case-34	>303.7 nmol/L	M+S	c.293-13 A/C>G	Homozygous
Case-35	298.0 nmol/L	M+S	Large Deletion (Promotor to Exon 3 of CYP21A2 gene) & c.1069C>T p.Arg357Trp	Compound heterozygous
Case-36	>291.4 nmol/L	M+S	c.293-13 A/C>G & E6 cluster variants	Compound heterozygous
Case-37	>291.4 nmol/L	M+S	c.293-13 A/C>G	Homozygous
Case-38	>291.4 nmol/L	M+S	c.293-13 A/C>G	Homozygous
Case-39	>291.4 nmol/L	M	c.293-13 A/C>G, c.332_339del 8bp Del & c.-113G>A	Homozygous
Case-40	118.9 nmol/L	S	c.293-13 A/C>G	Homozygous
Case-41	>300.9 nmol/L	M	c.293-13 A/C>G, c.332_339del 8bp Del & c.-113G>A	Homozygous
Case-42	>300.9 nmol/L	M	c.293-13 A/C>G	Homozygous
Case-43	208.1 nmol/L	M+S	c.955C>T p.Gln319Ter	Homozygous
Case-44	181.7 nmol/L	M+S	c.293-13 A/C>G	Homozygous
Case-45	>306.4 nmol/L	M+S	c.293-13 A/C>G	Homozygous
Case-46	89.6 nmol/L	S	c.40G>T p.Ala14Ser & c.955C>T p.Gln319Ter	Compound heterozygous
Case-47	79nmol/L	S	c.332_339del 8bp Del and c.-113G>A	Homozygous
Case-48	64nmol/L	M+S	c.293-13 A/C>G and c.332_339del 8bp Del	Compound heterozygous
Case-49	64nmol/L	M+S	c.293-13C/A>G	Homozygous
Case-50	64nmol/L	M+S	c.332_339del 8bp Del	Homozygous
Case-51	79nmol/L	M+S	Large Deletion (Promotor to Exon 7 of CYP21A2 gene) & c.1069C>T	Compound heterozygous
Case-52	64nmol/L	M+S	c.293-13C/A>G	Homozygous
Case-53	64nmol/L	M+S	c.293-13C/A>G	Homozygous
Case-54	64nmol/L	M+S	No varinat Detected	NA

Supplementary Table S1: Clinical data of affected newborns: Screening 17-OHP Levels and CYP21A2 Mutations

NA: Not applicable

M: MLPA was used to identify the variants/Deletions

S: Sanger sequencing was used to identify the variants

M+S: Both MLPA and Sanger sequencing was used.
